# Heat Treatment Effects for Controlling Dye Molecular States in the Hydrophobic Core of Over-1000 nm Near-Infrared (NIR-II) Fluorescent Micellar Nanoparticles

**DOI:** 10.1021/acsomega.1c05771

**Published:** 2022-02-08

**Authors:** Masakazu Umezawa, Hisanori Kobayashi, Kotoe Ichihashi, Shota Sekiyama, Kyohei Okubo, Masao Kamimura, Kohei Soga

**Affiliations:** Department of Materials Science and Technology, Faculty of Advanced Engineering, Tokyo University of Science, 6-3-1 Niijuku, Katsushika, Tokyo 125-8585, Japan

## Abstract

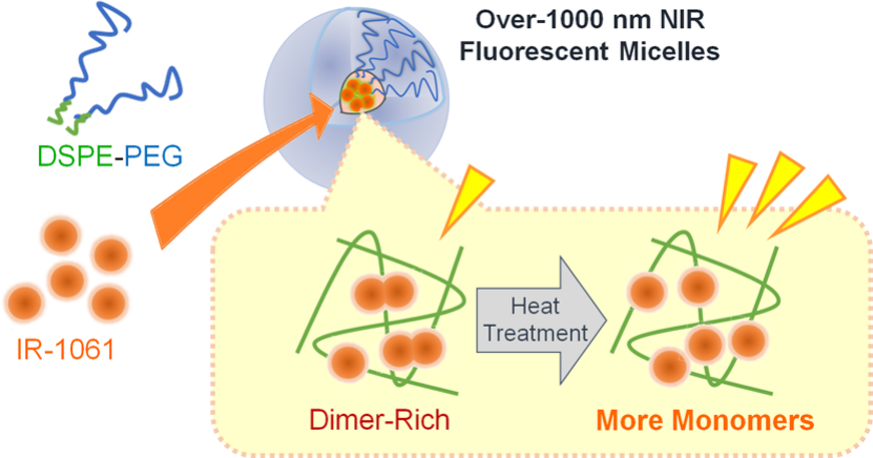

Organic molecules that emit near-infrared (NIR) fluorescence at wavelengths above 1000 nm, also known as the second NIR (NIR-II) biological window, are expected to be applied to optical *in vivo* imaging of deep tissues. The study of molecular states of NIR-II dye and its optical properties are important to yield well-controlled fluorescent probes; however, no such study has been conducted yet. Among the two major absorption peaks of the NIR-II dye, IR-1061, the ratio of the shorter wavelength (900 nm) to the longer one (1060 nm) increased with an increase in the dye concentration in tetrahydrofuran, suggesting that the 900 nm peak is due to the dimer formation of IR-1061. Both absorption peaks are also observed when IR-1061 is encapsulated in the hydrophobic (stearyl) core of micellar nanoparticles (MNPs) of a phospholipid–poly(ethylene glycol). The dimers in the MNP cores decreased *via* dimer dissociation by enhancing the mobility of the hydrophobic stearyl chains by heat treatment of the dye-encapsulating MNPs at 50–70 °C. The MNPs maintained the dissociated IR-1061 monomers in the core after recooling to 25 °C and showed a higher NIR-II fluorescence intensity than those before heat treatment. This concept will provide better protocols for the preparation of NIR-II fluorescent probes with well-controlled fluorescence properties.

## Introduction

Fluorescence *in vivo* imaging is a technique for visualizing biological structures and dynamic phenomena in real time using fluorescent probes introduced into the body. Light in the over-thousand-nanometer (OTN) near-infrared (NIR) region, also called the second NIR (NIR-II) biological window,^1^ is highly transparent to biological tissues,^2^ as it is less scattered by biological tissues than visible and shorter-wavelength NIR lights^3^ and is not strongly absorbed as in the mid-infrared wavelength region. Thus, fluorescence imaging in the OTN-NIR range is suitable for *in vivo* observation at depths on the order of centimeters.^4,5^ Previous studies have shown that OTN-NIR fluorescence is useful for imaging deep biological structures, such as the gastrointestinal tract,^6^ cancer lesion model,^7−12^ internal organs,^13^ brain vasculature,^14−17^ hindlimb blood flow,^18−21^ and whole-body vasculature^22−26^ in deeper parts of mice. OTN-NIR fluorescent probes have been developed, including rare-earth-doped ceramic nanoparticles,^5,6,14,22^ single-walled carbon nanotubes,^7,8,10,15,20,23,24,27^ quantum dots,^16^ and potentially more biocompatible nanomaterials composed of polymer conjugates with organic NIR-II fluorescent dyes^17,26^ as well as nanosized polymer micelles encapsulating the dyes.^11,12,18,21,28,29^ These nanomaterials of polymers with organic dyes provide biodegradable^25,30^ or renally clearable^11,17,18^ NIR-II fluorescence probes.

**Figure 1:**
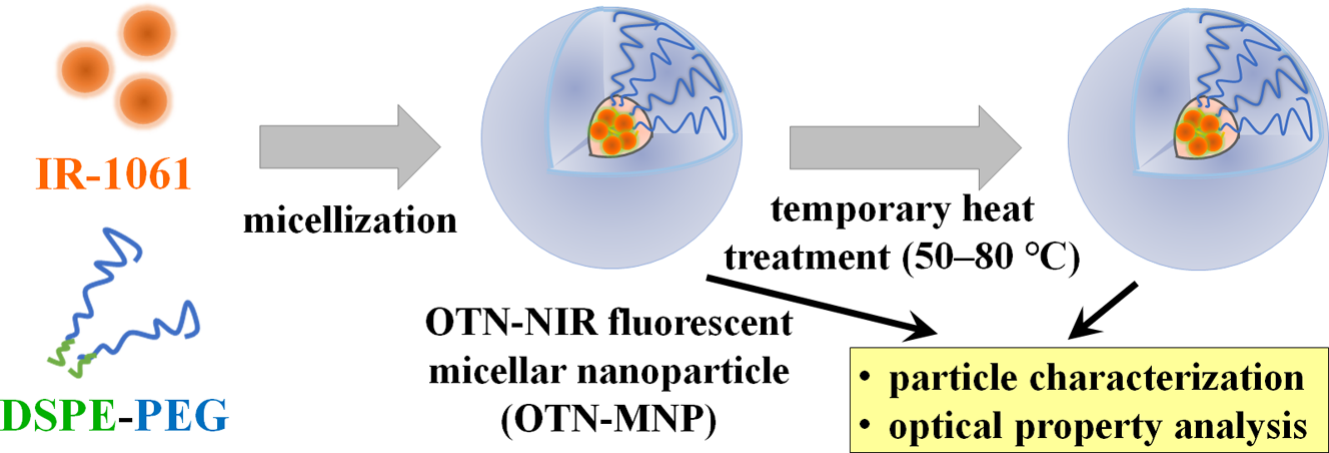
Schematic illustration of the protocol to prepare OTN-MNPs using a PL–PEG (DSPE-PEG) and IR-1061 dye and to investigate their optical properties.

IR-1061 is a polymethine dye that emits OTN-NIR fluorescence but is quenched when it interacts with water.^26,29^ It is necessary to design a probe structure that suppresses the quenching of IR-1061 for application in fluorescence imaging in physiological environments. Therefore, a structure has been proposed in which IR-1061 is loaded onto a hydrophobic core coated with a hydrophilic shell that maintains its dispersion in a hydrophilic environment.^18,21^ For example, micellar nanoparticles (MNPs) of a phospholipid (PL) derivative with a hydrocarbon chain at one end of the biocompatible hydrophilic polymer, poly(ethylene glycol) (PEG), encapsulating IR-1061 can be used as a fluorescent bioimaging probe.^18^ However, analysis and control of the molecular state and optical properties, possibly related to the fluorescence intensity of IR-1061 in the hydrophobic core, have been unsolved issues. The aim of this study was to investigate the molecular state of the NIR-II dye, IR-1061, and its changes in OTN-NIR fluorescent MNPs (OTN-MNPs) prepared by encapsulating the dye in PL–PEG micelles by analyzing their optical property profiles (Figure 1).

## Results and Discussion

### Absorption Spectra of IR-1061 Dissolved in Tetrahydrofuran at Different Concentrations

**Figure 2:**
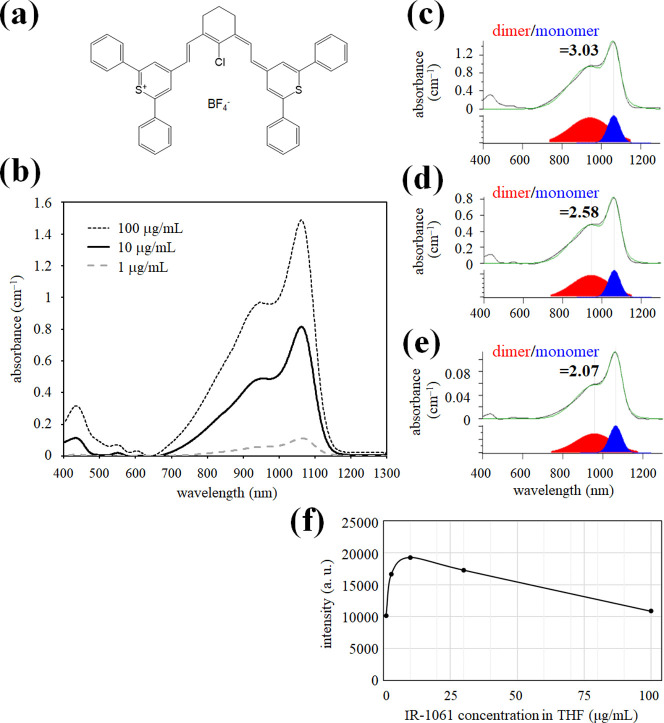
Concentration-dependent change in the absorption spectra of IR-1061 in THF. (a) Chemical structure of IR-1061, which has hydrophobicity and intramolecular polarization. (b) Absorption spectra of IR-1061 dissolved in THF at different concentrations. The peaks separated by Gaussian fitting for the data of IR-1061 solution at (c) 100 (d) 10, and (e) 1 μg/mL. (f) Concentration dependence of luminescence intensity (wavelength: 1050–1400 nm) of IR-1061 in THF.

IR-1061 is a hydrophobic molecule that is insoluble in water and slightly intramolecularly polarized due to its ionizing chemical structure (Figure 2a). Because of the presence of this polarity, IR-1061 can form dimers like aggregates to counteract its polarity in highly hydrophobic environments. We hypothesized that IR-1061 may form multiple molecular states in the solution. To investigate this hypothesis, we prepared a solution of IR-1061 in tetrahydrofuran (THF) at different concentrations and analyzed its absorption spectra. The IR-1061 solution in THF showed two peaks at 930 and 1060 nm in its absorption spectrum (Figure 2b), where the former increased as the concentration increased. These results suggest that IR-1061 can form dimers at high concentrations in THF. The association of dye molecules to form dimers has been reported in highly concentrated solutions for other dye molecules.^31^ Our data suggest that IR-1061 also forms dimers in high concentration solutions, as shown by the change in its absorption spectrum. Gaussian fitting of these spectral data separates the peaks and indicates that the ratios of the absorption at shorter wavelength (peaked at 930 nm) to that at the longer wavelength (peaked at 1060 nm) were 2.07, 2.58, and 3.03 in 1, 10, and 100 μg/mL solutions, respectively (Figure 2c–e). The concentration dependence of OTN-NIR fluorescence intensity of IR-1061 showed the dye’s quenching along with its dimer formation at a high concentration range (>5 μg/mL) (Figure 2f). Meanwhile, the concentration quenching of IR-1061 is also observed in other solvents such as acetonitrile (ACN), which has a higher solubility for IR-1061 than THF and starts its concentration quenching in a higher concentration range (unpublished data).

### Effect of Heat Treatment on Absorption Spectrum of IR-1061 in DSPE-PEG Micelles

**Figure 3:**
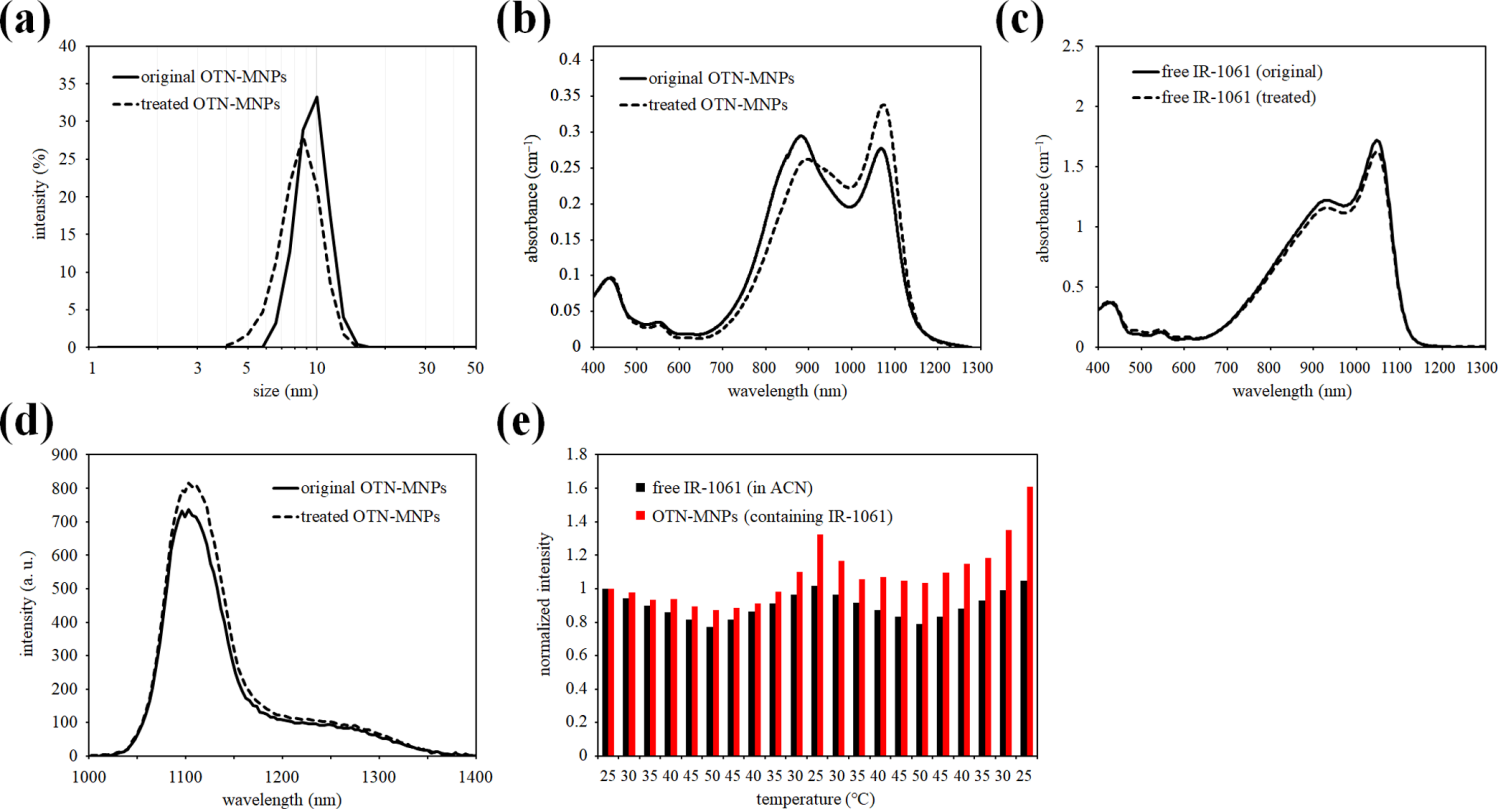
Effect of heat treatment of the size and optical properties of OTN-MNPs. The (a) size and (b–e) optical properties of OTN-MNPs (containing 12.5 μg/mL of IR-1061) before and after 2-cycle heating–cooling treatment between 25 and 50 °C are shown. (a) Hydrodynamic diameters determined by DLS. (b,c) Absorption spectra of (b) OTN-MNPs dispersed in water and (c) free IR-1061 (100 μg/mL) dissolved in ACN before and after the heat treatment. (d) Spectra of OTN-NIR fluorescence (excitation 980 nm) of OTN-MNPs dispersed in water before and after the heat treatment. (e) Change in the integrated fluorescence intensity (1050–1400 nm, excitation 980 nm) of the OTN-MNPs and free IR-1061 during the heating–cooling treatment.

MNPs of *N*-(carbonyl-methoxypolyethyleneglycol 2000)-1,2-distearoyl-*sn*-glycero-3-phosphoethanolamine (DSPE-PEG)-encapsulating OTN-NIR fluorescent IR-1061 dye were synthesized *via* a simple “one-pot” method.^21^ Following experiments were performed using ACN as a solvent for preparing OTN-MNPs because it has a higher solubility for IR-1061. The OTN-MNPs prepared by DSPE-PEG showed a peak of hydrodynamic diameter at 8–10 nm (Figure 3a) as reported previously^18^ and emission at 1100 nm under excitation with 980 nm light. The OTN-MNPs, that is, IR-1061 encapsulated in DSPE-PEG micelles, also showed two peaks in the range of 700–1200 nm in their absorption spectra (Figure 3b). Interestingly, heat treatment at 50 °C altered the absorption spectra of OTN-MNPs (Figure 3b), but not that of free IR-1061 in ACN (Figure 3c).

Interestingly, the fluorescence intensity of the OTN-MNPs at 25 °C increased after heat treatment at 50 °C for 5 min (Figure 3d). In this experiment, the temperature of the samples (OTN-MNP in water or free IR-1061 in ACN) was increased by five degrees every 5 min (*i.e.*, 1 °C/min), while the emission was analyzed at each temperature. After the analysis of emission at 50 °C, while the sample was held at this temperature for 5 min, the temperature was lowered by five degrees every 5 min. First, the emission intensity of free IR-1061 showed a temperature-dependent change, as generally seen in organic fluorescent dyes. In the range of 25–50 °C, the fluorescence intensity of IR-1061 decreased by 1.12% per 1 °C increase (Figure 3e). The temperature-dependent change in the fluorescence intensity of free IR-1061 was reversible, and the absorption spectra of free IR-1061 were not affected by the heat treatment (Figure 3e). These results indicate that IR-1061 does not degrade in this temperature change and that its emission intensity decreased with an increase in temperature because of the increased chance of energy transfer by collision with other molecules.

The pattern of the temperature-dependent change in the emission intensity of IR-1061 encapsulated in DSPE-PEG MNPs is different from that of the free IR-1061 solution in ACN. As the temperature of the OTN-MNP dispersion increased from 25 to 50 °C, the OTN-NIR fluorescence intensity decreased. When the temperature of the OTN-MNPs was returned to 25 °C, the fluorescence intensity was higher than that of the original OTN-MNPs dispersed in water (Figure 3e). The emission intensity of the OTN-MNPs further increased when the sample was heated and then cooled. Actually, the enhancement of the fluorescence intensity was up to 1.61-fold by the two-cycle heat treatment (Figure 3e) and not the level of several-fold increase. The reason for this limitation is that the dimer of IR-1061 also fluoresces weakly and that not all the dimers are completely dissociated into monomers by heating inside the hydrophobic core of OTN-MNPs, which has a limited volume. Anyway, unlike free IR-1061 (Figure 3c), the absorption spectrum of OTN-MNPs encapsulating IR-1061 in the hydrophobic core of DSPE-PEG micelles was affected by the temporary heat treatment (Figure 3b). Heat treatment promoted the dissociation of IR-1061 dimers into monomers in the hydrophobic core of the OTN-MNPs. The results of spectral analysis (Figure 3b) suggested that the dissociated monomers were retained in the MNP core even after the sample temperature reached 25 °C.

**Figure 4:**
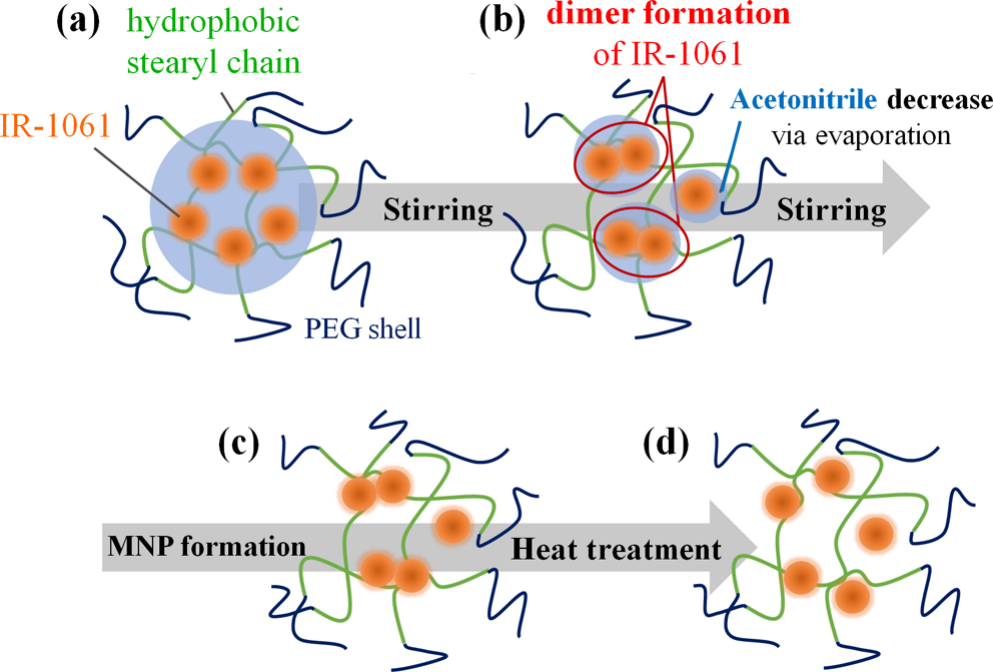
Potential mechanism of dimer formation and dissociation of IR-1061 in the MNP core during preparation followed by heat treatment. During evaporation of ACN (a,b), the IR-1061 is concentrated in ACN and in the hydrophobic core of MNPs (b) and forms dimers (c). The IR-1061 monomers were generated in the MNP core by dissociation of the dimers when the mobility of the hydrophobic chains of the MNPs and IR-1061 molecules increased. The dissociated monomers were trapped by the hydrocarbon chains and retained in the core after the temperature reached 25 °C (d).

These results suggest that in the MNP preparation process, the dye accumulates in a hydrophobic microenvironment just before the organic solvent is completely evaporated. Because IR-1061 has a slight intramolecular polarity compared to the hydrocarbon chain (stearyl group) that forms the hydrophobic core of MNPs of PL–PEG, the dye initially forms much dimer in the hydrophobic core. When the IR-1061-encapsulating MNPs were heated, the increased molecular mobility promoted the diffusion of IR-1061 and its dissociation into the monomer. Because the dissociated monomers could be trapped in the hydrophobic (hydrocarbon) chains and prevented from redimerization in the MNP core, they were retained in the core even after the temperature was lowered (Figure 4). Similar to this IR-1061, also in the case of aggregation-induced emission dyes, heat treatment may improve the dyes’ dispersion in microenvironment and change their optical properties, if the dye molecules tend to aggregate in the microenvironment that is composed of molecules with mismatched affinity for the dye.

### Effect of Treatment Temperature on the Molecular State of IR-1061 in DSPE-PEG Micelles

**Figure 5:**
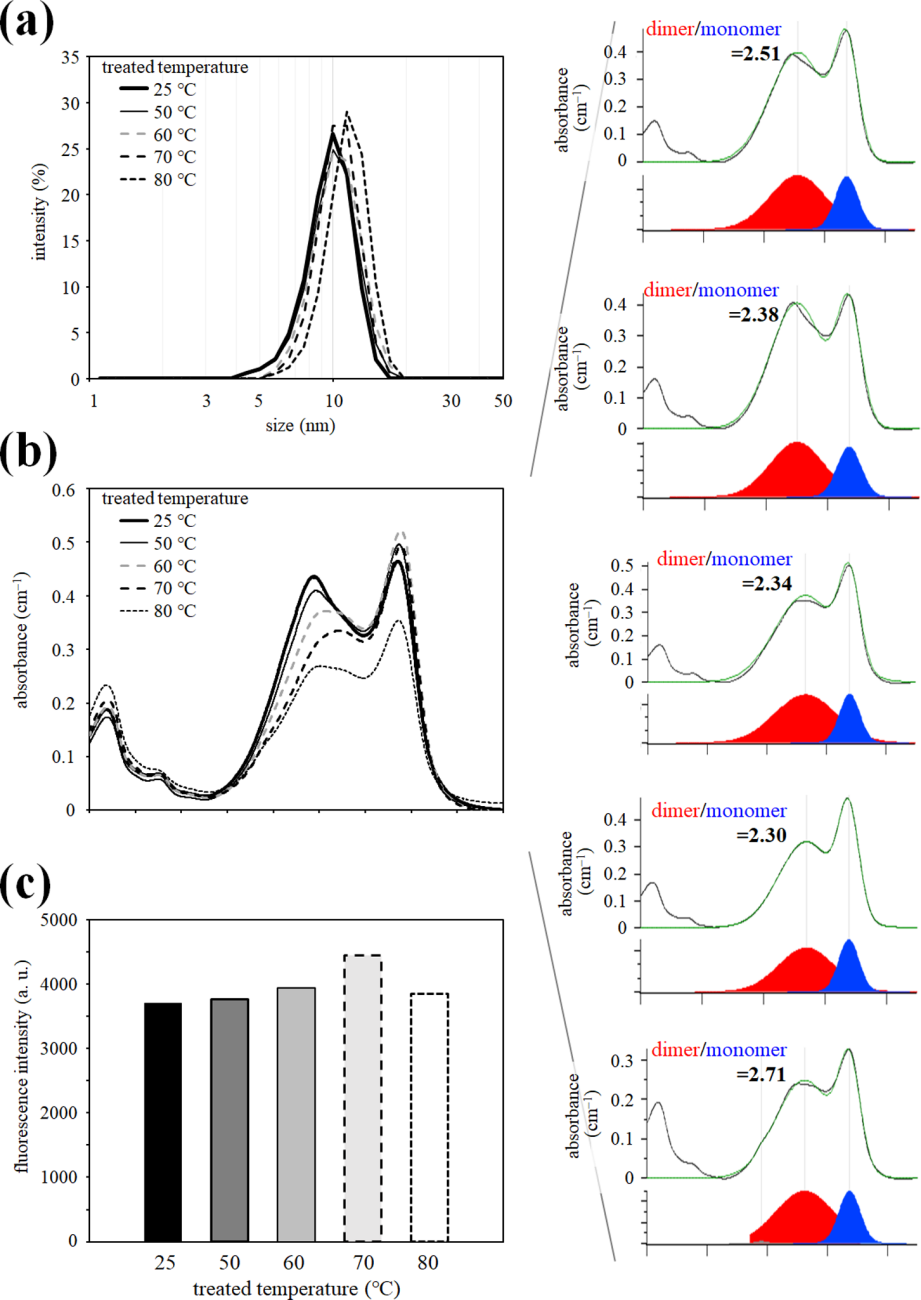
Effect of heat treatment with different temperatures (50–80 °C) on the size and optical properties of OTN-MNPs measured after reaching 25 °C. Effects of temporary heat treatment on (a) hydrodynamic diameters determined by DLS, (b) absorption spectra, and (c) intensity of OTN-NIR fluorescence (1050–1400 nm, excitation 980 nm) are shown. The measurements were performed for the OTN-MNPs (containing 12.5 μg/mL of IR-1061) at 25 °C, after the heat treatment at indicated temperatures for 5 min followed by reaching 25 °C. The right panel showed the peaks separated by Gaussian fitting for the absorption spectral data of samples after the heat treatment.

Furthermore, the effect of the treatment temperature on the properties of OTN-MNP was investigated. In this experiment, the temperature of the sample was increased from 25 °C to the indicated temperature (50, 60, 70, or 80 °C) at a rate of 5 °C/min, kept at the temperature for incubation for 5 min, and then cooled back to 25 °C at the same rate of temperature change. The effects of heat treatment were examined using a different protocol from Figure 3 to demonstrate the results to reach a conclusion with reproducibility and reliability. After the entire heating and recooling process, the absorption and OTN-NIR emissions were analyzed. The size of the OTN-MNPs was not affected by the heat treatment at any temperature (50–80 °C), as shown in Figure 5a. Temporary heat treatment altered the absorption spectrum (Figure 5b) and OTN-NIR fluorescence intensity (Figure 5c) of the OTN-MNP samples. The ratio of dimer to monomer of IR-1061 decreased in the OTN-MNPs after the treatment at 50, 60, and 70 °C (Figure 5b, right panel), which increased the fluorescence intensity of the OTN-MNPs. The absorption derived from IR-1061 decreased in the sample treated at 80 °C (Figure 5b), probably due to partial leakage of IR-1061 from the MNP core by disruption of the MNP structure at 80 °C. Therefore, the emission of the sample incubated at 80 °C was lower than that of samples treated at 60 and 70 °C (Figure 5c).

### *In Vivo* OTN-NIR Fluorescence Imaging of Tumor-Bearing Mice

**Figure 6:**
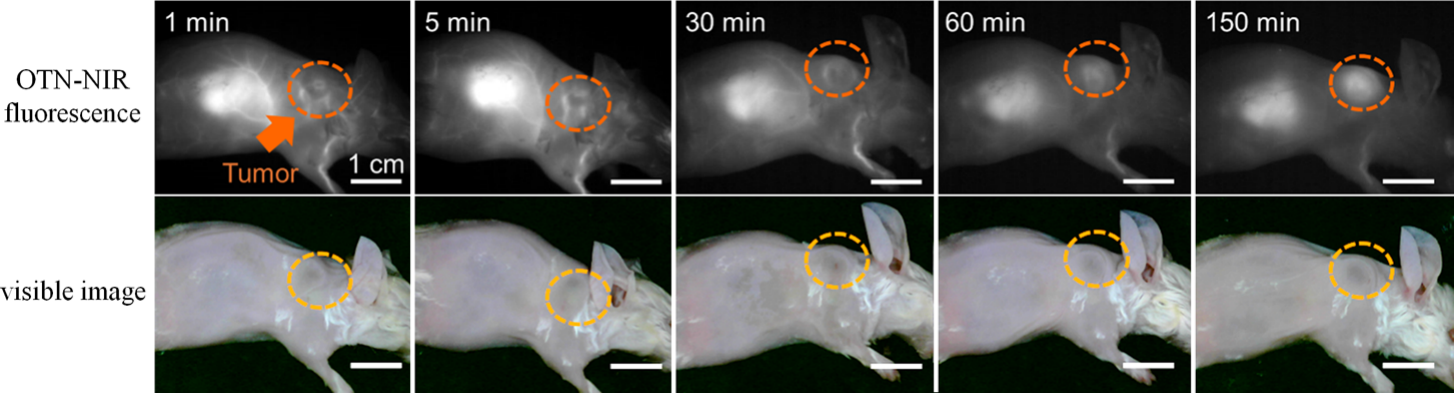
Imaging tumor under the skin by intravenous injection of the OTN-MNPs. After visualizing blood vessels and the liver at 1–30 min after injection, the OTN-MNPs distributed and visualized cancer lesion grown under the skin. Scale bars indicate 1 cm.

Finally, we prepared cancer-bearing mice to investigate the potential of OTN-NIR fluorescence live imaging of cancer by using OTN-MNPs after heat treatment. The OTN-MNPs incubated at 70 °C for 5 min were dispersed in physiological saline and injected into mice *via* the tail vein. The OTN-NIR fluorescence images showed that the OTN-MNPs visualized the normal blood vessels, blood-rich liver, and newly formed blood vessels around the cancer model from <1 min after the injection. At 60 and 150 min after the injection, the cancer lesion model was successfully visualized (Figure 6), possibly owing to the enhanced permeability and retention effect^32−34^ of the OTN-MNPs in cancer-bearing mice. These results indicate that the emissions of the heat-treated OTN-MNPs can be used to observe the deep tissues of mice. The enhancement of their fluorescence intensity by heat treatment is just up to several tens of percent in *in vitro* measurements, as shown in Figures 3 and 5, which did not lead to a significant improvement yet in *in vivo* imaging performance. This paper rather clarified the important finding of the molecular state of an OTN-NIR fluorescent dye, IR-1061, inside the micellar hydrophobic core. Further investigations are needed to find the best protocol, including the effective number of heating cycles at optimum temperature, to improve the imaging performance of targets in *in vivo* deep tissues.

## Conclusions

In this study, we investigated the concentration- and temperature-dependent changes in the optical properties of an OTN-NIR fluorescent dye, IR-1061, to reveal its molecular states when encapsulated in MNPs of PL–PEG. The ratio of the two absorption peaks of IR-1061 at 900 and 1060 nm was altered by its concentration in THF. The ratio of the peak at a shorter wavelength (900 nm) to the longer one increased with an increase in the dye concentration, suggesting that the short wavelength peak is attributed to the dimeric association of dye molecules. Spectral changes *via* dimer formation of IR-1061 were also observed when the dye was encapsulated in the hydrophobic core of MNPs composed of PL–PEG. In the MNP preparation process, the dye accumulates in a hydrophobic microenvironment just before the organic solvent is completely evaporated. Furthermore, because IR-1061 has a slight intramolecular polarity compared to the hydrocarbon chain (stearyl group) that forms the hydrophobic core of MNPs of PL–PEG, IR-1061 initially forms a dimer in the hydrophobic core. When the IR-1061-encapsulating MNPs are heated, the increased molecular mobility promotes the diffusion of IR-1061 and its dissociation into monomers, which is retained in the MNP hydrophobic core even after the temperature is lowered. The OTN-NIR fluorescent IR-1061-encapsulating MNP after heat treatment is applicable in *in vivo* imaging as it successfully visualized blood vessels and tumor tissue following intravenous injection into mice. The concept presented in this paper is expected to provide better protocols for the preparation of OTN-NIR fluorescent probes with well-controlled fluorescence properties.

## Experimental Section

### Materials

ACN and THF were purchased from Fujifilm Wako Pure Chemical Co. (Osaka, Japan). DSPE-PEG (Sunbright DSPE-020CN) was purchased from NOF Co. (Tokyo, Japan). IR-1061 and phosphate-buffered saline were purchased from Sigma-Aldrich Co. (MO, USA) and Otsuka Pharmaceutical Factory (Tokushima, Japan), respectively. All reagents were used without further purification.

### Preparation of MNPs Encapsulating IR-1061

The MNPs encapsulating IR-1061 were prepared using a previously described protocol^21^ with minor modifications. Briefly, DSPE-PEG_2k_ (1.5 mg) and IR-1061 (25 μg) were dissolved in 1 mL of ACN, followed by rapid addition of distilled water (2 mL); thus, the nominal concentration of IR-1061 for OTN-MNP preparation was set at 12.5 μg/mL in water. ACN was removed by evaporation to obtain an aqueous suspension of OTN-MNPs by stirring at 25 °C for 9 h (Figure 1). The obtained OTN-MNPs were purified by centrifuge filter purification (MWCO 10 kDa, 20 000*g*, 5 min, 3 times) and then dispersed in 2 mL of distilled water.

### Characterization of OTN-MNPs with Temperature Control

The hydrodynamic diameter of the OTN-MNPs was measured using a dynamic light scattering (DLS) particle size analyzer (LB-550; Horiba, Japan). The optical absorption spectra of the OTN-MNPs were measured using a UV–visible–NIR spectrometer V-770 (JASCO Co., Tokyo, Japan). Absorption spectral data containing multiple peaks were analyzed using Igor Pro software (Portland, OR, USA) for quantitative analysis of the area of each peak separated by Gaussian fitting. The NIR emission spectra of the OTN-MNPs were measured using a spectrometer (NIR-256-1.7, Avantes, Apeldoorn, Netherlands; integration time: 2 s) equipped with a temperature-controlled cuvette holder (qpod 2e; Quantum Northwest Inc., WA, USA) and a fiber-coupled laser diode (SP-976-5-1015-7; Laser Components, Olching, Germany) as the light source for 980 nm excitation (4.2 W). The temperature change was accomplished at a rate of 1 or 5 °C/min. The samples were held at the target temperatures (50, 60, 70, or 80 °C for each sample) for 5 min. The emissions were collected through a 1050 nm long-pass filter between the sample cuvette (PSK-10, Sansyo Co., Ltd., Tokyo, Japan) and a spectrometer detector. The emission intensities of the samples were recorded during the heating and recooling processes between 25 and 50 °C. The absorption spectra and the hydrodynamic diameter were also analyzed at 25 °C after heat treatment at 50–80 °C (Figure 1).

### OTN-NIR Fluorescence *in Vivo* Imaging

All experimental procedures involving animals were conducted in accordance with the national and institutional guidelines for the care and use of laboratory animals under the approval of the Animal Ethics Committee of Tokyo University of Science. Four-week-old female BALB/c mice were purchased from Japan SLC Co. (Hamamatsu, Japan) and fed the AIN-76A diet (Research Diets Inc., NJ. USA). After acclimation for 2 weeks, cultured murine colon cancer cells (Colon-26; 1.0 × 10^6^ cells) were subcutaneously injected into the backs of the mice. After 10 days, OTN-MNPs containing IR-1061 (0.5 mg/mL; dose 0.1 mL/mouse) were intravenously injected into the mice *via* the tail vein under anesthesia. The hair of the mice was removed before the OTN-MNP injection to avoid light scattering. The OTN-NIR fluorescence images were collected for mice with inoculated subcutaneous cancer lesions under 980 nm excitation using an OTN-NIR fluorescence *in vivo* imaging system (SAI-1000, Shimadzu, Kyoto, Japan).

## Abbreviations

ACN: acetonitrile
DLS: dynamic light scattering
DSPE-PEG: *N*-(carbonyl-methoxypolyethyleneglycol 2000)-1,2-distearoyl-*sn*-glycero-3-phosphoethanolamine
NIR: near-infrared
MNP: micellar nanoparticle
OTN: over-thousand-nanometer
OTN-MNPs: over-thousand-nanometer near-infrared fluorescent micellar nanoparticles
PEG: poly(ethylene glycol)
PL: phospholipid
THF: tetrahydrofuran
